# Comparative Analysis of the Fecal Microbiota of Relict Gull (*Larus relictus*) in Mu Us Desert (Hao Tongcha Nur) and Bojiang Haizi in Inner Mongolia, China

**DOI:** 10.3389/fvets.2022.860540

**Published:** 2022-04-06

**Authors:** Li Liu, Chao Du, Yunpeng Liu, Li Gao

**Affiliations:** Faculty of Biological Science and Technology, Baotou Teacher's College, Baotou, China

**Keywords:** gut microbiota, relict gulls, high-throughput sequencing, 16S rRNA gene, Mu Us Desert, Bojiang Haizi

## Abstract

The gut microbiota contributes to host health by improving digestive efficiency and maintaining homeostasis. The relict gull (*Larus relictus*), a national first-class protected bird in China, is listed as vulnerable in the International Union for Conservation of Nature Red List. Here, 16S rRNA gene sequencing was performed to characterize and compare the community composition and diversity of the gut microbiota sampled from relict gulls in two breeding sites. In total, 418 operational taxonomic units (OUTs) were obtained and classified into 15 phyla and 228 genera. Alpha diversity analysis revealed no significant differences in community diversity among the two breeding sites. Beta diversity analyses showed that the microbial communities at the two sites were different. Six dominant phyla and fourteen dominant genera were identified. The most abundant bacterial genera had a significant relationship with the diet and living environment, and some bacterial genera were found to adapt to the plateau environment in which relict gulls live, which enables the relict gulls to use local resources effectively to accumulate energy. Simultaneously, a variety of highly abundant pathogenic bacteria were found, suggesting that these gulls may spread diseases among the local gull population. Certain measures should be taken to protect this species and to prevent the spread of diseases.

## Introduction

The relict gull (*Larus relictus*) is a medium-sized gull that breeds in scattered locations in drought lakes in the Russian Far East, eastern Mongolia, and Kazakhstan. The largest breeding group is called the Ordos subpopulation, which is distributed in northern China in Shaanxi and Inner Mongolia (1). It is listed as vulnerable in the International Union for Conservation of Nature Red List and is a national first-class protected bird in China. There are approximately 10,000–19,999 relict gulls worldwide (2).

The Ordos subpopulation of relict gulls migrates over the Inner Mongolia Plateau and reaches the coast of the Bohai Sea for winter. Mu Us Desert (KX) and Bojiang Haizi (BJ) in Ordos City, Inner Mongolia Autonomous Region, China are both located on the Inner Mongolia Plateau and are important breeding grounds and stopover sites for relict gulls (3, 4). The relict gulls mainly live around sandy lakes and feed on sand flora and lake fauna. The habitats of the two breeding sites are different. Previous studies highlighted that habitat and dietary nutrition are both potential influencing factors of the animal gut microbial community (5–8), thus, the gut microbiota may also change accordingly.

The gut microbiome is a collection of all microbial cells and related genetic substances present in the host gastrointestinal tract. The intestinal microbiota is numerous and diverse and is closely related to various physiological functions of the host's body. Some types of gut microbiota are associated with metabolic diseases, such as obesity (9) and diabetes (10), and the occurrence and progression of gastrointestinal diseases (11–13). Others participate in functional processes that are critical for homeostasis (14, 15). In summary, the composition and diversification of the gut microbiota are closely associated with the health of host. To date, studies on the gut microbiota of avian species have focused on the influence of particular bacteria or bacterial pathogens on the host (16, 17). The rapid development of high-throughput 16 S rRNA gene sequencing technology has enriched our knowledge of gut microbiota diversity. It is widely used for quick and efficient characterization of the gut microbial communities of many organisms, such as humans (18), cows (19), pigs (20), chickens (21), and a selected number of avian species (22–25).

Characterization of the avian gut microbial community contributes to the knowledge of avian microbiology and will likely advance efforts to protect the living environments of wild birds. Moreover, the identification of pathogenic bacteria harbored by wild birds helps to prevent the spread of related diseases. At present, neither there is any related research on relict gull gut microbiota nor that of different breeding grounds. In the present study, fecal samples of relict gulls from two different breeding grounds were collected. Then, the bacterial microbiota composition and differences in relict gulls across the different sites during the breeding period were determined. Analysis of the bacterial microbiota composition and its relation to the breeding environment has great significance for the physiological ecology and population management of relict gulls. The findings of the present study can provide insight into the gut microbial composition of relict gulls and provide a theoretical basis for their protection, such as disease prevention and control.

## Materials and Methods

### Ethics Statement

The current study was performed in accordance with the suggestions on animal care and ethics in China. Non-invasive techniques (26) were used to collect fecal samples. The Animal Ethics and Welfare Committee of Baotou Teachers College approved the implementation of the project, and the management authorities of KX and BJ of Ordos City in Inner Mongolia approved the sampling of relict gull fecal samples.

### Study Subjects and Areas

The study subjects were relict gulls in the KX in Wushen Banner, Ordos City and BJ in Dongsheng District, Ordos City, Inner Mongolia Autonomous Region, China. The KX and BJ are both important breeding grounds and stopover sites for relict gulls (3, 4).

The KX (107° 20 E to 111°30 E; 37° 27.5 N to 39° 22.5 N), one of the four largest sandy areas in China, is located in the Ordos Plateau and has a semi-arid, mid-temperate continental monsoon climate. The samples were collected in the Wushen Banner, which is located in the middle of the KX, at an altitude of 1,300–1,400 m. The annual average temperature is 6.8°C, the annual sunshine duration is 2,800–3,000 h, and the annual precipitation is 350–400 mm. The annual breeding season of the relict gulls in the KX is from early April to the end of July.

Bojiang Haizi is located in the west of Dongsheng District (109° 14 E to 109° 23 E; 39° 45 N to 39°52 N) and has a semi-arid, mid-temperate continental monsoon climate. BJ samples were collected in the nature reserve at an altitude of 1,367–1,412 m. The average annual temperature is 5.2°C, the annual sunshine duration is 3,200 h, and the annual average precipitation is 325.8 mm. It is the most concentrated distribution area and the most important breeding ground for the Ordos population of relict gulls. BJ is comprised of inland wetlands, where the relict gulls mainly live around lakes and feed on sand plants and lake creatures.

### Sample Collection

A total of 13 relict gulls' fecal samples were collected, of which 6 samples were collected from BJ (109° 17 E to 109°18 E; 39° 47 N to 39° 48 N) and 7 samples were collected from the KX (108° 39 E to 108° 58 E; 39° 11 N to 39° 12.5 N). Fresh and clean fecal samples were collected from the two sites in June to ensure uniformity of the sampling period. Generally, relict gulls leave feces around their nests. Fresh feces were sampled after the relict gulls are left. The samples were immediately collected into 5 ml sterile centrifuge tubes. To minimize possible contamination from the ground, only the upper layer of the feces was collected. The collected fresh fecal samples were placed on dry ice, transported to the laboratory, and stored at −80°C until further analysis.

### DNA Extraction

DNA was extracted using the E.Z.N.A. Stool DNA Kit (D4015, Omega, Inc., GA, USA), according to the manufacturer's instructions, as described (27). Generally, total DNA was isolated and eluted in elution buffer and used for polymerase chain reaction (PCR), the amplified region was the V3–V4 hypervariable region of the bacterial 16S rRNA gene, the used primers were 341F (5′-CCTACGGGNGGCWGCAG-3′) and 805R (5′-GACTACHVGGGTATCTAATCC-3′). The PCR products were then purified and quantified. The amplicon pools were prepared for sequencing. The libraries were sequenced using the NovaSeq 6000 platform (Illumina) in accordance with the manufacturer's protocol.

### Data Analysis

Paired-end reads were assigned to the samples based on their unique barcodes and truncated by cutting off the barcode and primer sequences. Paired-end reads were merged using Flash software. Raw reads were quality-filtered using specific filtering conditions to obtain high-quality clean reads using fqtrim (v0.94). UCLUST was a program used to group all the sequences into operational taxonomic units (OTUs) at an identity threshold of 97% similarity. Chimeric sequences were filtered using VSEARCH software (v2.3.4). Singleton OTUs (OTUs with only one sequence) were removed from all the datasets. Based on the information extracted from the SILVA database (version 138), each OTU was assigned to the lowest possible taxonomic level based on a minimum bootstrap threshold of 80%. The OTU table was subsampled accordingly to adjust the sampling depth using the “multiple_rarefactions.py” program from the Quantitative Insights Into Microbial Ecology (QIIME) pipeline. Alpha- and beta-diversities were calculated based on the *de novo* taxonomic tree constructed by the representative chimera-checked OTU set using FastTree. OUT-level α-diversity indices, such as abundance-based coverage estimator (ACE), Chao1, Shannon index, and Simpson index, were calculated using the OTU table in QIIME to evaluate the richness and diversity of the bacteria. Beta diversity was determined using non-metric multidimensional scaling (NMDS) based on unweighted UniFrac distance matrices. Linear discriminant analysis (LDA) effect size (LEfSe) analysis was used to indicate the significant ranking of rich components in the two breeding grounds (28). Briefly, LEfSe analysis, with an LDA threshold of > 4, used the non-parametric factorial Kruskal-Wallis sum-rank test, and then the (unpaired) Wilcoxon rank-sum test was used to identify the most differentially abundant taxa. Figures were plotted using the R software (29) with “ggplot2” package. The functions of the bacteria with significant abundance (>1% in each group) were predicted using the Kyoto Encyclopedia of Genes and Genomes (KEGG) database.

## Results

### Sequence Statistics

**Figure 1:**
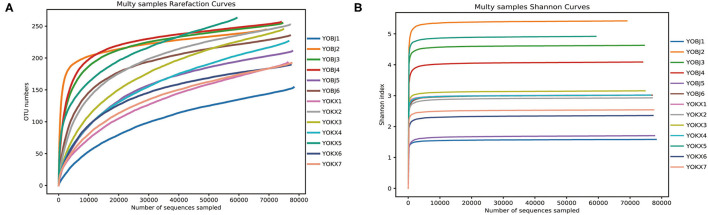
The Rarefaction curve **(A)** and Shannon diversity curve **(B)** of the bacterial community in the analyzed samples. YOBJ, relict gull (*Larus relictus*) of Bojiang Haizi; YOKX, relict gull (*Larus relictus*) of Mu Us Desert.

From 13 fecal samples across the two study areas, a total of 1,015,717 effective sequences were obtained, and 72,424–79,387 (mean 78,132 ± 1,824) effective sequences (mean length was 423 bp) were obtained for each sample. At a sequence similarity level of 97%, 418 OTUs were obtained, and the mean number of OTUs in each sample was 225 ± 34 (range: 154–264). The OTUs were classified into 15 phyla, 23 classes, 68 orders, 129 families, and 228 genera. Both the rarefaction curve and the Shannon index curve were almost flat and indicate that a plateau in the bacterial diversity curve and deeper sequencing had no significant influence on microbial diversity (Figure 1).

**Figure 2:**
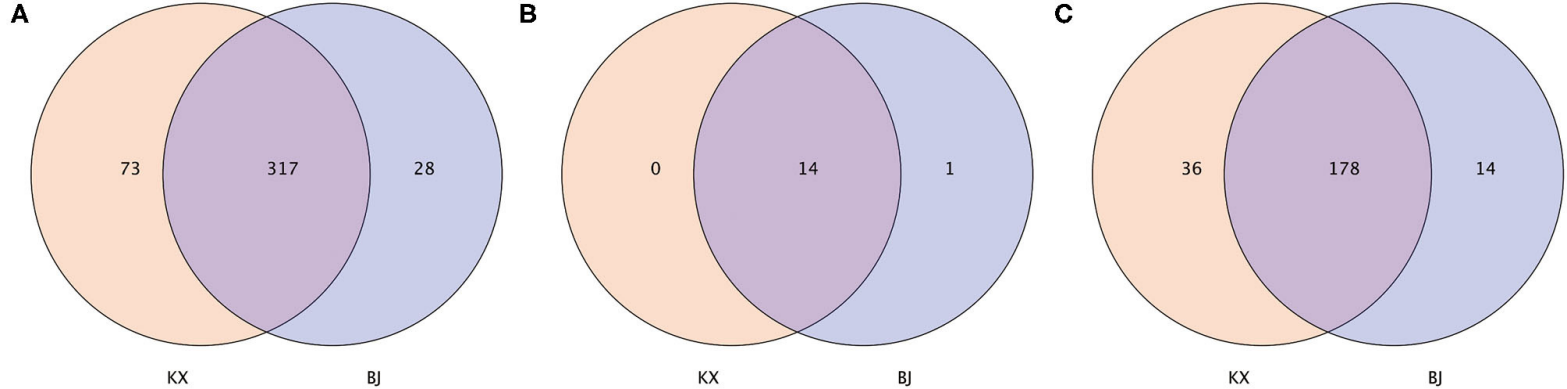
Venn diagram of the bacterial community in the analyzed groups. It shows the numbers of shared or not shared operational taxonomic units (OTUs; 97% sequence identity) **(A)**, phyla **(B)**, and genera **(C)** by Mu Us Desert (KX) and Bojiang Haizi (BJ) individuals depending of overlaps.

In terms of OTUs, 317 OTUs were shared by relict gull individuals from the two areas. There were 73 OTUs present only in the gut microbiota of relict gulls in the KX, whereas 28 OTUs were present only in the gut microbiota of relict gulls in BJ (Figure 2A). Fourteen phyla were shared by relict gull individuals in the two areas (Figure 2B). There was one more gut microbiota in relict gulls in BJ, Kiritimatiellaeota, which did not exist in KX. Finally, the gut microbiota composition of the different birds was analyzed. The number of genera shared between the two sites was 178, and there were 36 genera present only in the gut microbiota of relict gulls in KX and 14 only in BJ (Figure 2C).

### Alpha Diversity and Beta Diversity Analysis

**Figure 3:**
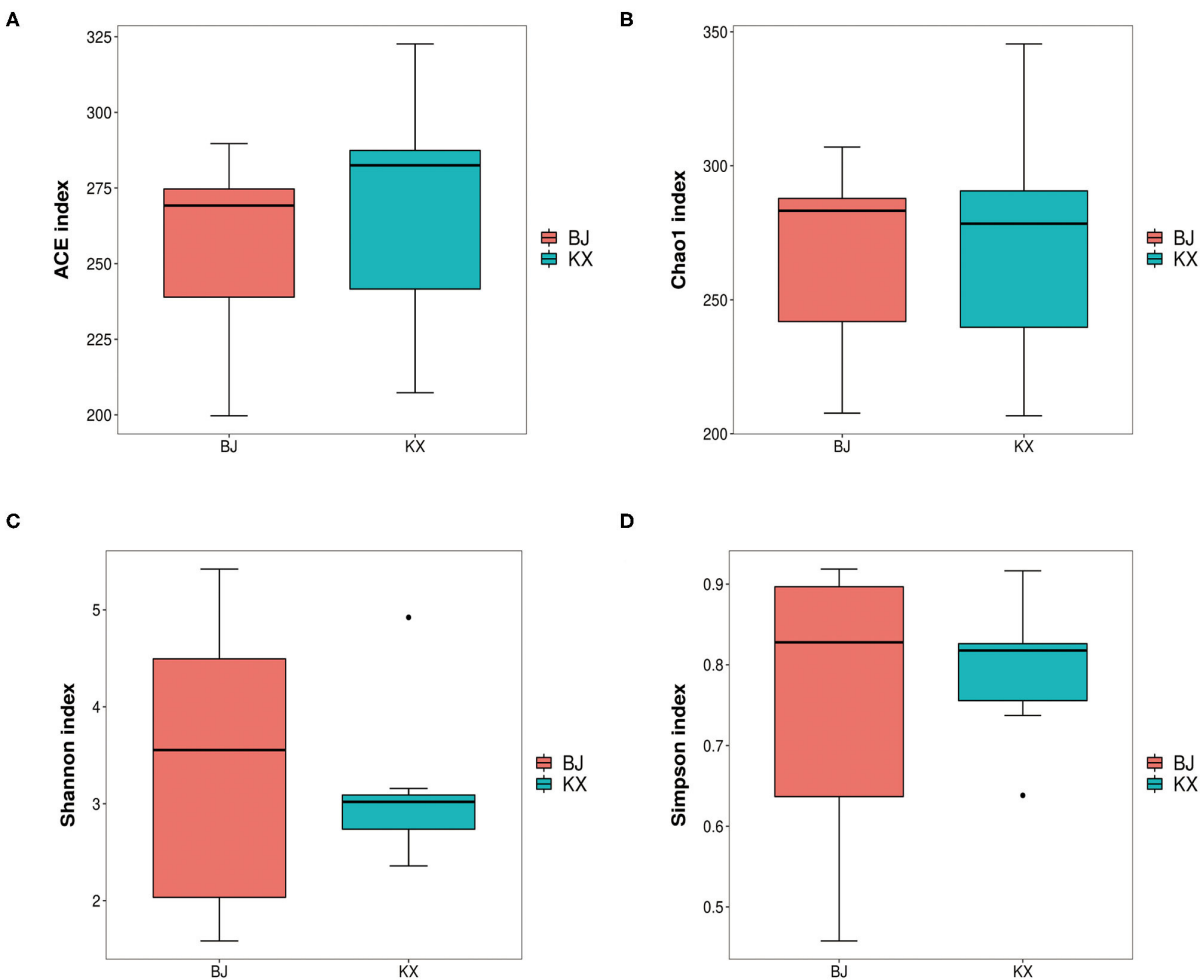
Abundance-based coverage estimator (ACE) index **(A)**, Chao1 diversity **(B)**, Shannon diversity **(C)**, and Simpson indices **(D)** of bacterial populations in each sample. BJ, Bojiang Haizi; KX, Mu Us Desert.

In the current study, results of alpha diversity analysis revealed no significant differences in ACE (255.53 ± 33.79 and 267.21 ± 38.95), Chao1 (266.43 ± 38.99 and 270.19±45.58), Shannon (3.41 ± 1.57 and 3.14 ± 0.84), and Simpson (0.75 ± 0.19 and 0.79 ± 0.09) indices between BJ and KX (*p* > 0.5; Figure 3).

**Figure 4:**
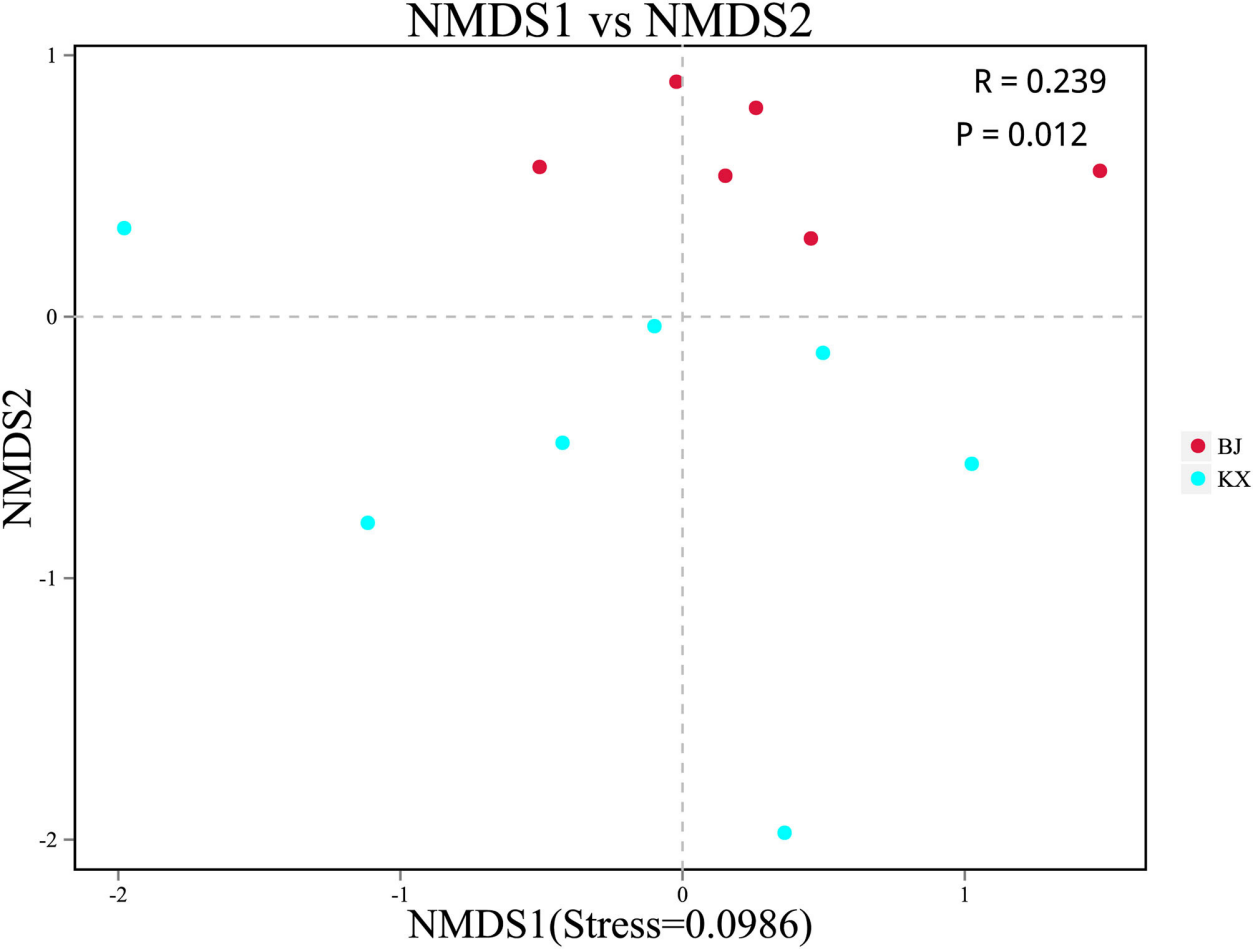
Non-metric multidimensional scaling (NMDS) analysis. Each point represented one sample. The distance between points represented the degree of difference. The closer the distance between the samples in the graph, the higher the similarity between them. BJ, Bojiang Haizi; KX, Mu Us Desert.

Beta diversity analysis was performed to determine whether differences existed in bacterial composition between the KX and BJ. Scatter plots were generated using NMDS based on the unweighted UniFrac distance. The analysis showed a significant clustering trend of fecal samples of birds from the same area, suggesting that there were significant differences in the microbial community composition between relict gulls from the two sites (Figure 4).

### Global Composition of Gut Bacterial Communities of the Two Sites

**Figure 5:**
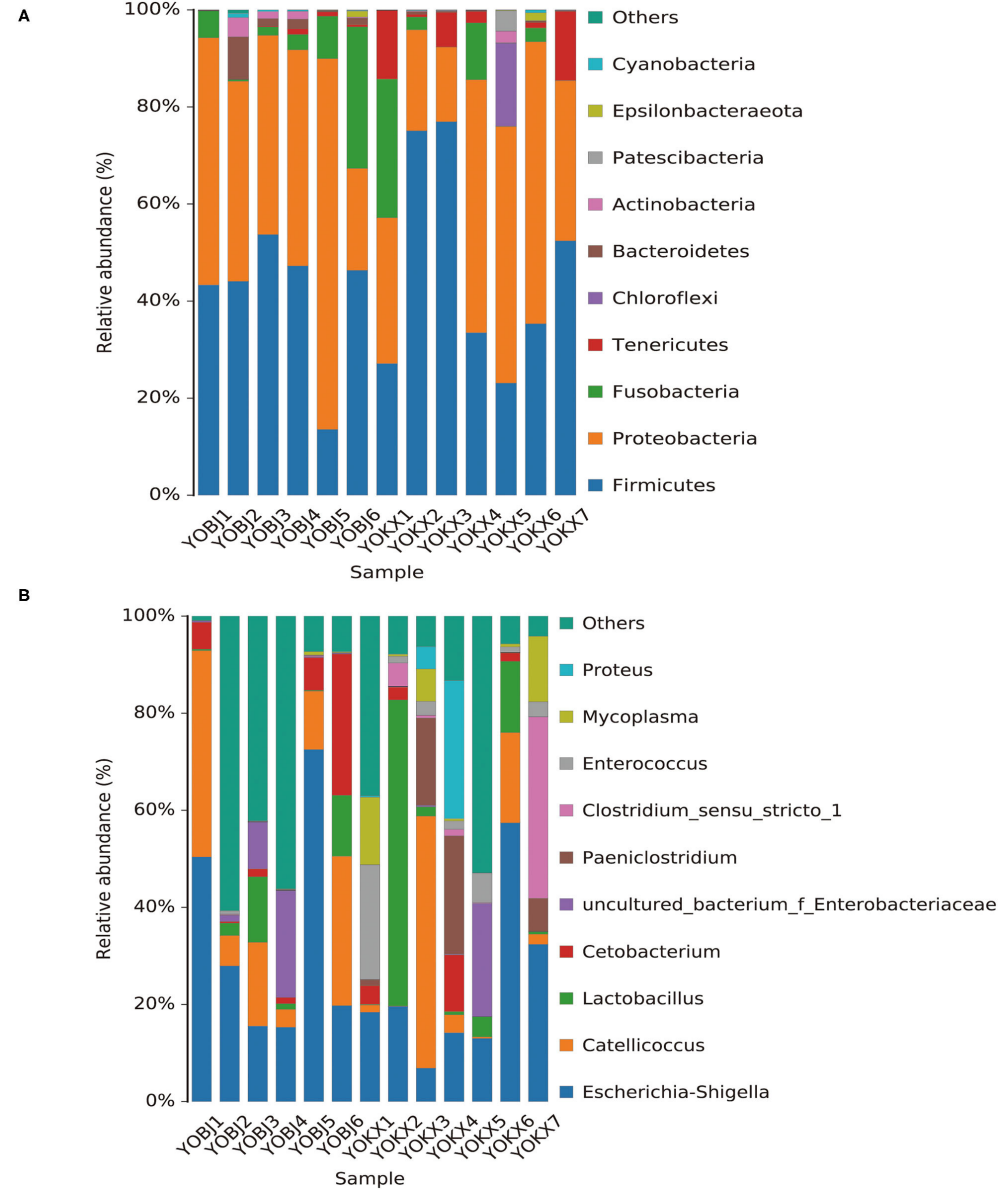
Bar graph of the relative bacterial abundance at phylum **(A)** and genus **(B)** levels. The Bacterial those with relative abundance (%) over 1% were shown. Others are bacterial with a relative abundance of <1%. BJ, Bojiang Haizi; KX, Mu Us Desert.

The bacterial composition of the fecal samples was analyzed. At the phylum level, 15 phyla were identified in the 13 fecal samples, of which six showed an average relative abundance above 1% (Figure 5A): Firmicutes, Proteobacteria, Fusobacteria, Tenericutes, Bacteroidetes, and Chloroflexi. The cumulative proportion of these six phyla was 98.33% for each sample. The gut microbiota of relict gulls in the KX was dominated by Firmicutes, whereas the dominant phylum in BJ was Proteobacteria.

The bacterial composition of the fecal samples at the genus level was also analyzed. A total of 228 bacterial genera were identified in the 13 fecal samples, of which 14 showed an average relative abundance above 1%: *Escherichia-Shigella, Catellicoccus, Lactobacillus, Cetobacterium, uncultured_bacterium_f_Enterobacteriaceae, Paeniclostridium, Clostridium_sensu_stricto_1, Enterococcus, Mycoplasma, Proteus, Fusobacterium, Candidatus_Arthromitus, Sporosarcina*, and *Plesiomonas* (Figure 5B). The dominant genus in both BJ and KX was *Escherichia-Shigella*.

### Differences Between Gut Microbial Communities of Different Sites

**Figure 6:**
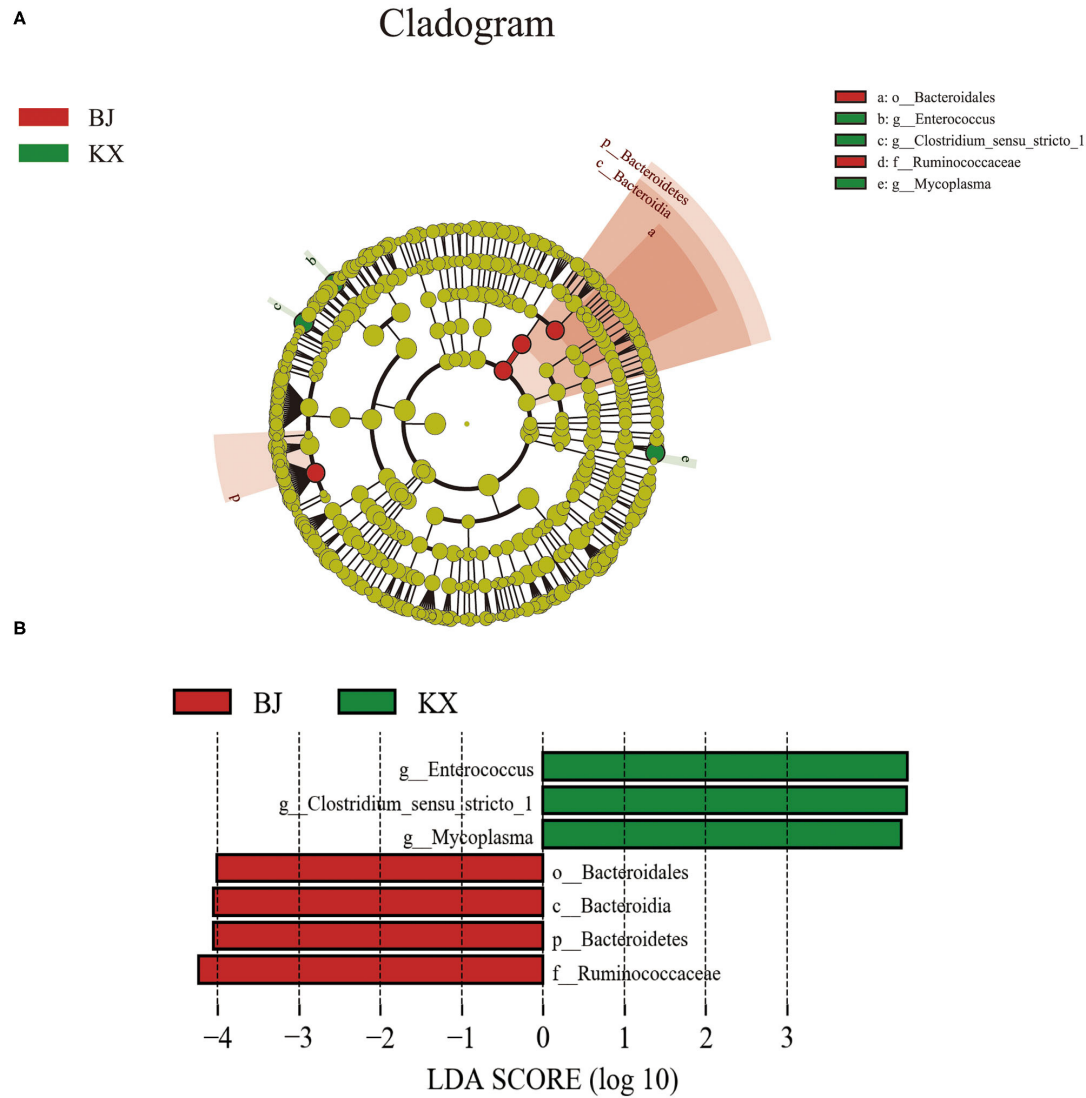
Linear discriminant analysis (LDA) effect size (LEfSe) analysis. **(A)** The microbial species had significant differences in the two breeding sites. Different colors presents different groups, the species classification at the level of genus, family, order, class, and phylum were exhibited from the outside to the inside. **(B)** The plot from LEfSe analysis. The length of the bar column represents the LDA score. The figure exhibited the microbial with significant differences between the two breeding grounds (LDA score > 4.0).

The composition of the gut microbiota of birds in different areas was investigated. The results indicate that there were significant differences in the composition of the gut microbiota between the different breeding sites of relict gulls (Figure 6A). The LEfSe analysis results (Figure 6B) indicate that the microorganism with significant differences between the two sites was Bacteroidetes (LDA > 4.0, *p* < 0.05). At the genus level, the biomarkers that demonstrated significant differences between the two areas were *Enterococcus, Clostridium*, and *Mycoplasma* (LDA > 4.0, *p* < 0.05).

### Bacterial Community Functional Prediction

**Figure 7:**
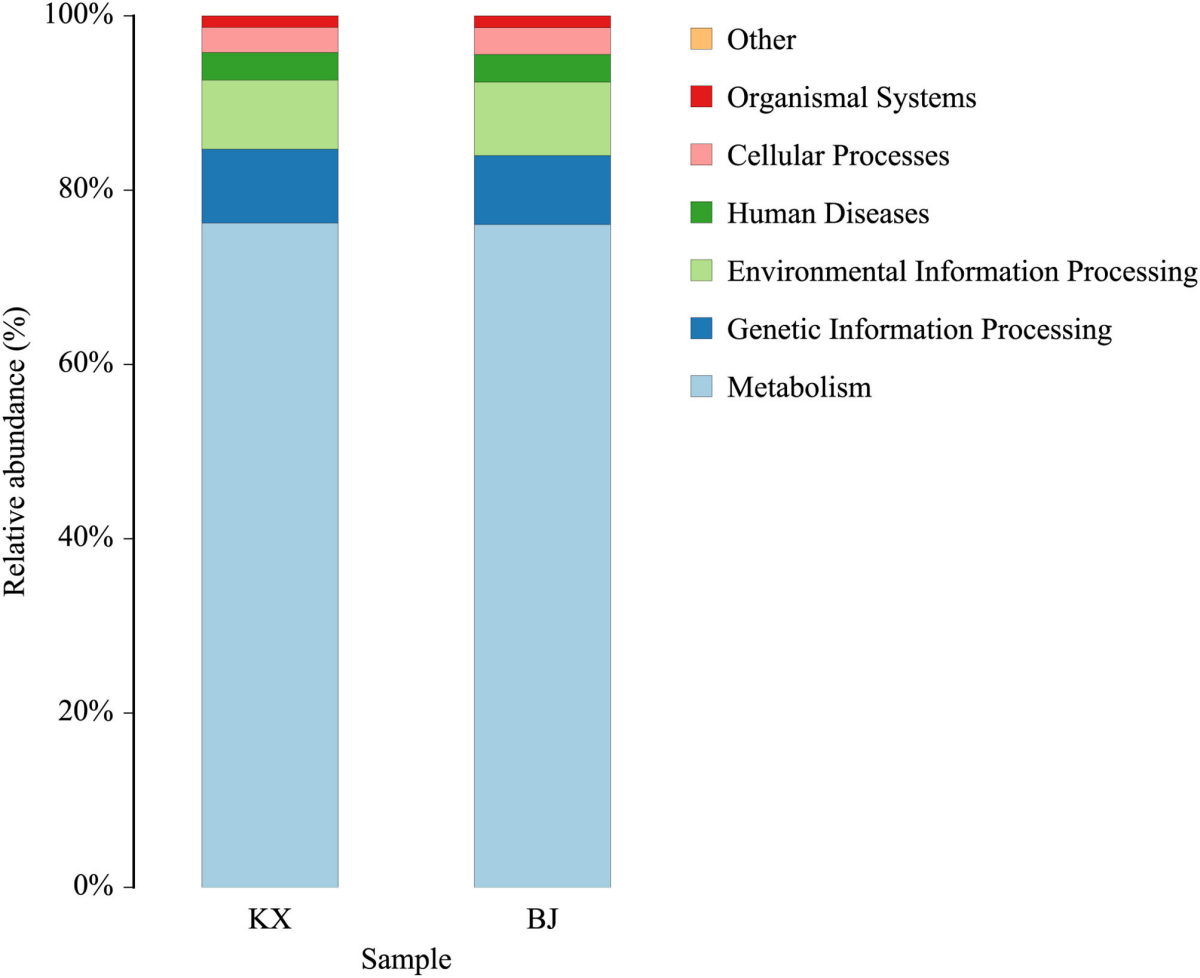
Bacterial community functional prediction by Kyoto Encyclopedia of Genes and Genomes (KEGG) database.

The functions of the bacteria with significant abundance (>1% in each group) in the relict gulls at the two sites were predicted using the KEGG database. The results show that the main functions of these bacteria were cellular processes, environmental information processing, human diseases, metabolism, and organismal systems (Figure 7).

## Discussion

The relict gull is a vulnerable species that relies heavily on dry lakes in Asia (30). The Ordos subpopulation of relict gulls migrates over the Inner Mongolia Plateau and reaches the coast of the Bohai Sea for winter (3, 31). Ordos Wetland, including the KX and BJ, are both vital stopover sites and breeding grounds. The habitats of the two breeding sites are different. Previous studies have highlighted that habitat and dietary nutrition are potential influencing factors of the animal gut microbial community (5–8). At the same time, the gut microbiota plays important roles in energy intake and metabolism, immune homeostasis maintenance, and the reproductive health of the host (32). Therefore, it is important to know the composition of the intestinal bacterial community to aid assessment of the breeding environment and determine whether sufficient food is available to the species living there. However, to date, there have been no studies on the intestinal microbiota of relict gulls. In the present study, 16S rRNA gene high-throughput sequencing technology was used to detect differences in the bacterial microbiota of relict gull (*Larus relictus*) in different places.

In terms of alpha diversity, no difference in the abundance and diversity of OTUs was observed between the relict gulls of the KX and BJ (BJ). Beta diversity was represented by NMDS based on unweighted UniFrac distance and demonstrated that there were significant differences in the gut microbiota composition between the two groups. This indicates that different habitat environments affected the gut microbial communities.

At the phylum level, 15 phyla were identified in the 13 fecal samples, and Firmicutes, Proteobacteria, Fusobacteria, Tenericutes, Bacteroidetes, and Chloroflexi showed an average relative abundance above 1%. Firmicutes and Proteobacteria were the most abundant microbiota in the relict gulls at both sites. This is consistent with the results of previous studies on other avian species (27, 33). These phyla exist in multiple avian species, indicating that they are important functional bacteria in the gastrointestinal tract of relict gulls.

The presence of these phyla in the gut microbiota of multiple avian species indicates that these bacteria might play important functional roles in the gastrointestinal tract of relict gulls, such as digestion, absorption of nutrients, and immune response. Firmicutes and Proteobacteria are both key metabolic phyla in the gut and can break down compound sugars, fatty acids, and polysaccharides in foods, generating energy for animals to absorb and accumulate (34–36). Fusobacteria play important roles in the production of butyrate, which in turn accelerates fat accumulation in the body and strengthens immunity (37). Tenericutes are widespread in the environment and are opportunistic pathogenic bacteria (33). Furthermore, Bacteroidetes are considered to be related to chronic intestinal inflammation (38). Chloroflexi is abundant and ubiquitous freshwater microbes (39). These results show that relict gull breeding in the two sites may be at risk of gastrointestinal disease. Local government departments should take certain measures to protect the health of the breeding relict gulls.

The habitat environment has an important effect on gut microbial communities. Kiritimatiellaeota was present in relict gulls in BJ but not in KX. Kiritimatiellaeota mediates arginine biosynthesis and regulates fatty acid synthesis. It is often found in plateau animals (40) and is a strategy for plateau animals to use low-fat food to provide energy in order to adapt to extreme environments. BJ has an altitude of 1,367–1,412 m, which is higher than that of the KX (1,300–1,400 m), but the difference is not significant. The annual average temperature of BJ is 5.2°C, which is lower than that in the KX, but is not significantly different. Thus, we speculate that the presence of Kiritimatiellaeota may be a consequence of the difference in microbial communities between the two sites. However, the dominant plant species in these two sites have not yet been identified, thus, the plants consumed by the relict gull Ordos subpopulation at the two study sites are unknown. Hence, further study is required to establish the consumption behaviors of the relict gull Ordos subpopulation.

Additionally, together Firmicutes and Proteobacteria were the dominant bacterial phyla at both sites. However, Firmicutes were the more dominant gut microbiota of relict gulls in the KX, whereas the dominant phylum in BJ was Proteobacteria. The results of LEfSe analysis indicate that at the phylum level, the relative abundance of Bacteroidales was significantly higher in BJ than in KX. Previous studies have reported that an increased Firmicutes/Bacteroidetes ratio is related to obesity in humans (41, 42) and strengthens the capacity to harvest energy from their diet (43). In this study, relict gulls in the KX had a higher Firmicutes/Bacteroidetes ratio than those in BJ (BJ), indicating that relict gulls in the KX had an enhanced capacity to promote food energy intake and maintain metabolic equilibrium.

In previous studies, abundant bacterial genera were considered to have derived from the food sources and the wider environment. For example, *Catellicoccus* was highly abundant in various gulls' fecal samples (44–46), indicating that it may be a symbiont or associated with their special diets and lifestyle. *Cetobacterium* is a bacterium that is commonly found in the intestinal tract of fish (47), *Candidatus_Arthromitus* is a bacterium genus that is commonly found in insect intestines (48). Critically, fish and insects are likely important to the diet of relict gulls. Therefore, these bacteria may have colonized the gull's intestines through the food consumed. Uncultured_bacterium_f_Enterobacteriaceae, *Enterococcus, Paeniclostridium, Mycoplasma, Plesiomonas*, and *Proteus* are all dominant bacterial genera in the soil and water environment (49–53), indicating that environmental factors have an important influence on the gut microbiota of relict gulls.

At the same time, two genera of probiotics were found in the dominant bacteria *Lactobacillus* and *Sporosarcina*. *Lactobacillus* and *Sporosarcina* have been previously reported to be beneficial bacteria in the intestine, capable of anti-inflammatory activities and protection against pathogens (54, 55). Therefore, the presence of these two types of bacteria can help relict gulls to resist disease. However, most of the highly abundant bacteria found were potentially pathogenic bacteria, such as *Paeniclostridium, Clostridium_sensu_stricto_1, Enterococcus, Mycoplasma, Proteus*, and *Plesiomonas*, which have been associated with diseases in multiple species (49–51, 53, 56, 57). Through functional prediction, these bacteria were found to be related to human diseases. This result proves that the relict gulls in KX and BJ carry pathogenic microorganisms that threaten the health of humans and other animals. Therefore, certain measures should be taken to prevent the spread of avian zoonotic diseases.

## Conclusion

In summary, in the current study, high-throughput sequencing was performed to analyze fecal samples of relict gull (*Larus relictus*) breeding in the KX and BJ in Inner Mongolia, China. The analysis revealed that the gut microbiota differed among different breeding sites. We propose that the most abundant bacterial genus had a significant relationship with the diet and living environment. Moreover, the presence of some bacterial genera may be due to adaptation to the plateau environment in which relict gulls live. This would enable the relict gulls to effectively use local resources and accumulate energy. Simultaneously, a variety of highly abundant pathogenic bacteria were found, suggesting that these individuals are at risk and may spread diseases. Certain measures should be taken to protect this species and to prevent the spread of avian zoonotic diseases. This study provides a theoretical basis for relict gull protection and related disease prevention and control.

## Data Availability Statement

The datasets presented in this study can be found in online repositories. The names of the repository/repositories and accession number(s) can be found below: https://www.ncbi.nlm.nih.gov/bioproject/PRJNA788023.

## Ethics Statement

The animal study was reviewed and approved by the current study was performed in accordance with the suggestions on animal care and ethics in China. Non-invasive techniques were used to collect fecal samples. The Animal Ethics and Welfare Committee of Baotou Teachers College approved the implementation of the project, and the management authorities of KX and BJ of Ordos City in Inner Mongolia approved the sampling of relict gull fecal samples.

## Author Contributions

LG and LL designed the research. LG, YL, and LL collected the samples. CD analyzed the data. LG wrote the manuscript. LG, LL, CD, and YL revised the manuscript. All authors contributed to the article and approved the submitted version.

## Funding

This work was funded by the Inner Mongolia Natural Science Foundation (No. 2021BS08013), the Baotou Teachers College High-level Research Achievement Cultivation Project (No. BSYKJ2021-ZQ03), and the High-level Talents Introduced Scientific Research Startup Fund Project of Baotou Teacher's College (No. BTTCRCQD2020-003).

## Conflict of Interest

The authors declare that the research was conducted in the absence of any commercial or financial relationships that could be construed as a potential conflict of interest.

## Publisher's Note

All claims expressed in this article are solely those of the authors and do not necessarily represent those of their affiliated organizations, or those of the publisher, the editors and the reviewers. Any product that may be evaluated in this article, or claim that may be made by its manufacturer, is not guaranteed or endorsed by the publisher.

## Abbreviations

OTUs: operational taxonomic units
KX: Mu Us Desert
BJ: Bojiang Haizi
NMDS: non-metric multidimensional scaling
LDA: linear discriminant analysis
LEfSe: linear discriminant analysis (LDA) effect size
KEGG: Kyoto Encyclopedia of Genes and Genomes
PCR: polymerase chain reaction.
